# The Effects of Two Nrf2 Activators, Bardoxolone Methyl and Omaveloxolone, on Retinal Ganglion Cell Survival during Ischemic Optic Neuropathy

**DOI:** 10.3390/antiox10091466

**Published:** 2021-09-15

**Authors:** Jia-Ying Chien, Yu-Yau Chou, Jhih-Wei Ciou, Fang-Yun Liu, Shun-Ping Huang

**Affiliations:** 1 Institute of Medical Sciences, Tzu Chi University, Hualien 970, Taiwan; 100712016@gms.tcu.edu.tw; 2 Department of Molecular Biology and Human Genetics, Tzu Chi University, Hualien 970, Taiwan; 104712123@gms.tcu.edu.tw (Y.-Y.C.); 107712111@gms.tcu.edu.tw (J.-W.C.); liufangyun0701@gms.tcu.edu.tw (F.-Y.L.); 3 Department of Ophthalmology, Tzu Chi University, Hualien 970, Taiwan

**Keywords:** ischemic optic neuropathy, nuclear factor E2-related factor 2, bardoxolone methyl, omaveloxolone, retinal ganglion cell, oxidative stress, nuclear factor kappa-light-chain-enhancer of activated B cells

## Abstract

Nonarteritic anterior ischemic optic neuropathy (NAION) is one of the most common acute optic neuropathies that affect the over 55-year-old population. NAION causes the loss of visual function, and it has no safe and effective therapy. Bardoxolone methyl (methyl 2-cyano-3,12-dioxooleana-1,9(11)-dien-28-oate; CDDO-Me; RTA 402) is a semisynthetic triterpenoid with effects against antioxidative stress and inflammation in neurodegeneration and kidney disease that activates the nuclear factor erythroid 2-related factor 2 (Nrf2) signaling pathway. Moreover, RTA 402 is an FDA-approved compound for the treatment of solid tumors, lymphoid malignancies, melanoma, and chronic kidney disease. Omaveloxolone (RTA 408) is an activator of Nrf2 and an inhibitor of NFκB, possessing antioxidative and anti-inflammatory activities in mitochondrial bioenergetics. RTA 408 is also under clinical investigation for Friedreich ataxia (FA). In this study, a rodent anterior ischemic optic neuropathy (rAION) model induced by photothrombosis was used to examine the therapeutic effects of RTA 402 and RTA 408. Treatment with RTA402 results in antiapoptotic, antioxidative stress, anti-inflammatory, and myelin-preserving effects on retinal ganglion cell (RGC) survival and visual function via regulation of NQO1 and HO-1, reduced IL-6 and Iba1 expression in macrophages, and promoted microglial expression of TGF-β and Ym1 + 2 in the retina and optic nerve. However, these effects were not observed after RTA 408 treatment. Our results provide explicit evidence that RTA 402 modulates the Nrf2 and NFκB signaling pathways to protect RGCs from apoptosis and maintain the visual function in an rAION model. These findings indicate that RTA 402 may a potential therapeutic agent for ischemic optic neuropathy.

## 1. Introduction

Nonarteritic anterior ischemic optic neuropathy (NAION) is the most common acute optic neuropathy in adults and has no sex predilection [1]. NAION typically presents with acute, unilateral, painless loss of visual acuity associated with a visual field defect and optic disc swelling. Patients who experience an attack of NAION have a significant risk of a similar episode in their fellow eye [2], resulting in irreversible retinal ganglion cell (RGC) apoptosis in the retina [3], demyelination of the optic nerve [4], and disruption of the blood–optic nerve barrier [5]. The pathogenesis of NAION is unclear, and there is currently no effective treatment to reduce the damage caused by NAION. Notably, nocturnal systemic hypotension, hypertension, hyperlipidemia, and diabetes are the major conditions involved in the development of NAION [6,7,8,9].

Recently, neuroinflammatory components have been identified in both patients and rodent models [10], including macrophage infiltration and the breakdown of the retinal–blood barrier [11,12]. Microglia act as the first innate immune response in the central nervous system (CNS) [13]. During maturation, resting microglia are the predominant form in the brain. Resting microglia survey and maintain the microenvironment of the CNS, and under inflammatory conditions, these resting microglia convert into their activated form [14,15], express proinflammatory cytokines (including interleukin-1 (IL-1), IL-6, and nitric oxide (NO)), and produce oxidative stress, NFκB, and tumor necrosis factor-alpha (TNF-α); together, these events cause a decrease in the rigor of the optic nerve–blood barrier and enhancements of immune factor recruitment and peripheral immune cell infiltration, leading to neuronal damage [16,17,18,19,20]. In addition, microglia have a major regulatory role in CNS immunity and modulate the release of inflammatory cytokines for self-limitation to restrict other immune effector cells [21]. The modulation of activated microglia may be a candidate for ischemic optic neuropathy therapy.

Bardoxolone methyl (methyl 2-cyano-3,12-dioxooleana-1,9(11)-dien-28-oate; CDDO-Me; RTA 402) is a synthetic oleanane triterpenoid that activates nuclear factor erythroid 2-related factor 2 (Nrf2), which plays a crucial role in arresting NFκB and defending against oxidative stress and inflammatory activity [22]. Recently, a phase three clinical study showed that RTA 402 can increase the estimated glomerular filtration rate (eGFR) and defend against oxidative stress in patients with chronic kidney disease (CKD) via activation of the Nrf2 signaling pathway [23,24]. In addition, RTA 402 augments antioxidative responses in a mouse model of multiple sclerosis by Nrf2 activation, NFκB suppression, and the preservation of myelin, axons, and neurons in the CNS [25,26]. Omaveloxolone (N-(2-cyano-3,12-dioxo-28-noroleana-1,9(11)-dien-17-yl)-2-2-difluoropropanamide; RTA 408) is a synthetic oleanane triterpenoid that is also an Nrf2 activator. RTA 408 has antioxidative as well as anti-inflammatory effects and regulates mitochondrial bioenergetics. Previous research studies have indicated that RTA 408 increases Nrf2 expression to upregulate NQO1 and HO-1 in dermal diseases [27]. RTA 408 reduces the content of IL-1β, stimulates NFκB phosphorylation, and expresses Nrf2-targeted genes to attenuate MMP-9 expression in rat brain astrocytes, suggesting that it is beneficial for the treatment of neurodegenerative disorders [28]. This compound can also improve Friedreich ataxia (FA), a genetic neurodegenerative disorder, as it has shown safety and efficacy to rescue mitochondrial function in FA patients in a phase two study [29,30]. Together, the development of RTA 402 and RTA 408, targeting anti-inflammatory responses and antioxidative stress in molecular signaling pathways, may be beneficial for NAION therapy.

In this study, we chose the rodent anterior ischemic optic neuropathy (rAION) model to mimic the pathophysiology of ischemic neuropathy, including ischemia, optic disc edema, and inflammation [31,32,33]. We assumed that the potential neuroprotective targets RTA 402 and RTA 408 were involved in the modulation and prevention of visual function in ischemic optic neuropathy.

## 2. Materials and Methods

### 2.1. Animals

**Figure 1:**
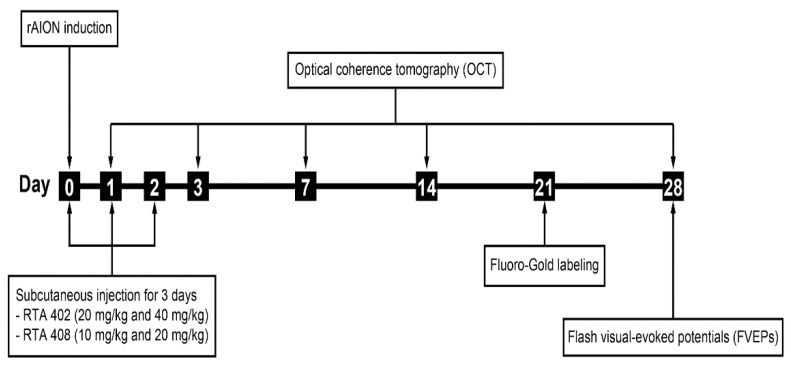
Flowchart of the experimental procedure for RTA 402 and RTA 408 treatment in rodent anterior ischemic optic neuropathy (rAION) model.

Male Wistar rats (75–100 g) were purchased from BioLASCO Taiwan Co., Ltd. (Taipei, Taiwan). All animal experiments were carried out under the guidelines of the Tzu Chi Institutional Animal Care and Use Committee (IACUC No. 106051). The rats were housed in cages with food and water under a 12 h day/night cycle. Animals were anesthetized using a mixture containing ketamine (100 mg/kg, Health-Tech Pharmaceutical Co., Ltd., Taipei, Taiwan) and xylazine (10 mg/kg, Health-Tech Pharmaceutical Co., Ltd., Taipei, Taiwan) In addition, 0.5% Alcaine eye drops (Alcon, Mechelen, Belgium) were used. Animals were divided into four groups; the number of rats used in this study is described in Table 1. The design of the experimental process is shown in Figure 1.

### 2.2. Administration of RTA 402 and RTA 408

RTA 402 (cat no: HY-13324, MedChemExpress LLC, Monmouth Junction, NJ, USA) and RTA 408 (cat no: HY-12212, MedChemExpress LLC) were diluted with a mixed solution of 0.9% normal saline and Tween 80 (9:1). Rats were subcutaneously administered one of the following treatments: RTA 402 once a day at one of two doses (20 mg/kg or 40 mg/kg) or vehicle after rAION model induction, or RTA 408 once a day at one of two doses (10 mg/kg and 20 mg/kg) or vehicle after rAION model induction.

### 2.3. rAION Induction

The rAION model was induced according to our previous steps. After anesthesia and pupil dilation, rose bengal (2.5 mM, 1 mL/kg, Sigma-Aldrich, St. Louis, MO, USA) was intravenously injected via the tail vein with a 27G needle. Within 1 min, the optic disc was treated with a 532 nm argon laser with a 500 μm spot size operated at 80 mW (MC-500 Multicolor laser, Nidek Co., Ltd., Tokyo, Japan), with 12 pulses each of 1 s duration under an OFA5.4 laser contact lens (Ocular Instruments Inc. Bellevue, WA, USA). The sham group was subjected to laser irradiation without rose bengal injection. The RTA 402 and RTA 408 solutions were subcutaneously injected into the rats immediately after rAION induction and administered once daily for three consecutive days.

### 2.4. Image-Guided Optical Coherence Tomography (OCT) Imaging System

The optic nerve width (ONW) and retinal nerve fiber layer (RNFL) were photographed by an image-guided spectral domain OCT system (Micro IV, Phoenix Research Labs, CA, USA). After anesthesia and pupil dilation, rats were placed on a platform, the optic nerve head (ONH) width of the middle of the optic nerve was measured by linear scan, and the RNFL was obtained by circular scan around the optic nerve. The width of Bruch’s membrane opening (BMO) was defined as the ONW. The fiber region from the ganglion cell layer (GCL) was measured as the RNFL. Each OCT scan was estimated using ImageJ software (version 1.8.0_172; U.S. National Institutes of Health, Bethesda, MD, USA, https://imagej.nih.gov/ij/, accessed on 26 March 2021).

### 2.5. Flash Visually Evoked Potential (FVEP) Recordings

The experimental procedure to acquire the FVEPs followed that of our previous reports [34,35]. In short, after anesthesia, electrodes were placed on the frontal area (bregma +1 mm) and occipital area (bregma 8 mm, lateral 3 mm) and reference electrodes were plated on the tail. The FVEPs were detected using a Celeris system (Diagnosys LLC, Lowell, MA, USA). The settings included turning the background illumination off, a ganzfeld flash intensity of 0 db, a single flash of light at 1.9 Hz, a test average of 100 sweeps, an artifact rejection threshold of 20 mV, and a sampling rate of 2000 Hz. The P1-N2 amplitudes were recorded for the FVEPs in all groups.

### 2.6. Retrograde RGCs with Fluoro-Gold (FG)

These procedures have been further explained in our previous studies [34,35]. At day 21 after rAION induction, rats were anesthetized and placed in a stereotactic apparatus (Stoelting, Wood Dale, IL, USA). The skull was exposed, and 2 μL of 5% FG was injected into the superior colliculus on each side via a Hamilton syringe. After one week, the eyeballs were obtained after the rats had been euthanized. The eyeballs were incubated in 10% formalin for 2 h. Each retina was dissected and flat-mounted on a slide. The morphometry of the RGCs was detected by fluorescence microscopy (Axio Scope A1, Zeiss, Oberkochen, Germany) with a filter set (excitation filter = 350–400 nm, emission filter = 515 nm) and photographed with a digital camera (Axiocam 305 color, Zeiss, Oberkochen, Germany). The number of RGCs at a distance of 1 or 3 mm from the ONH was counted for determination of the central and mid-peripheral RGC densities. Eight random regions per slide were imaged in both the central and mid-peripheral areas. RGC density was calculated by ImageJ software.

### 2.7. Retina and Optic Nerve Preparation

The rats were euthanized with 20–30% CO_2_ chamber per minute in a cage (5 L/min) after the VEP experiment, and the eyes were removed immediately. The eyeballs and segments of the optic nerve (5 mm) were fixed with 4% paraformaldehyde for 2 h and then transferred to 10%, 20%, and 30% sucrose overnight for gradient dehydration. Eyes were enucleated and embed with OCT Compound (SAKURA Finetek USA, Inc. Torrance, CA, USA) and kept at −80 °C for sectioning.

### 2.8. Terminal-Deoxynucleotidyl-Transferase-Mediated Nick End Labeling (TUNEL) Assay

The frozen retinal sections were placed on 8 μm slides. To ensure the use of equivalent fields for comparison, all frozen retinal sections were prepared with retinas taken at 1–2 mm from the ONH. The TUNEL reaction (Click-iT™ Plus TUNEL Assay for In Situ Apoptosis Detection, Invitrogen, Waltham, MA, USA) was performed to detect apoptotic cells by confocal microscopy (LSM900, Zeiss). TUNEL-positive cells in the RGC layer were counted in 10 high-powered fields (HPFs; 400 × magnification). Three sections per rat were averaged. To quantify the number of TUNEL-positive cells, the positive cells were counted using ImageJ software.

### 2.9. Immunohistochemistry (IHC)

The retinal sections with the optic nerve were washed with PBS and blocked with blocking buffer (1% bovine serum albumin (BSA), 1% normal goat serum, and 1% Triton X-100 in 1XPBS). The sections were incubated with primary antibodies against Iba1 (1:200, ab178846, Abcam, Cambridge, UK), IL-6 (1:200, ab9324, Abcam, Cambridge, UK), ED-1 (1:50, MCA341GA, Bio-Rad, Hercules, CA, USA), CNPase (1:200, ab6319, Abcam, Cambridge, UK) and Ym1 + 2 (1:50, ab192029, Abcam, Cambridge, UK). After labeling, the sections were immersed in PBS. Next, the sections were incubated with secondary-antibody-conjugated Alexa Fluor dyes (Invitrogen, Waltham, MA, USA) for 1 h. Counterstaining was performed using DAPI (1:500, Sigma, St. Louis, MO, USA). Sections were visualized and photographed with a Zeiss confocal microscope (LSM900, Zeiss, Oberkochen, Germany). For ED-1 and Ym1 + 2, the positive cells were quantified from six images at the lesion site of the optic nerve using ImageJ. For quantitative analysis of CNPase, the CNPase-stained area per DAPI molecule was estimated by ImageJ.

### 2.10. Immunoblot Analysis

Protein was extracted from the retina tissue, and a total of six samples from each group were prepared with RIPA lysis buffer containing protease and phosphatase inhibitors (Cat: 78442, Invitrogen, Waltham, MA, USA). The protein concentration was determined with a BCA protein assay kit (Cat: 23225, Thermo, Waltham, MA, USA). A total of 50 μg of protein was loaded onto 10% SDS-PAGE. After gel electrophoresis, the SDS-PAGE gels were transferred to 0.45 μm PVDF membranes. After blocking with blocking buffer (5% nonfat milk in 1x TBST) for 1 h, the blots were incubated with the primary antibodies Nrf2 (1:500, sc-722, Santa Cruz, Dallas, TX, USA), NQO1 (1:5000, ab80588, Abcam, Cambridge, UK), HO-1 (1:200, ab13243, Abcam, Cambridge, UK), IkBa (1:5000, ab32518, Abcam, Cambridge, UK), *p*-IkBa (#9246, Cell Signaling, Danvers, MA, USA), *p*-NFκB (1:200, ab86299, Abcam, Cambridge, UK), NFκB (1:500, ab16502, Abcam, Cambridge, UK), Arg1 (1:500, #93668, Cell Signaling, Danvers, MA, USA), and CD206 (1:500, ab64693, Abcam, Cambridge, UK) for 16 h at 4 °C. After washing the PVDF membrane with TBST buffer, the membranes were incubated with the secondary antibody conjugated to horseradish peroxidase (HRP) (1:10000, Bio-Rad, Hercules, CA, USA) at room temperature (RT) for 1 h. Each membrane was detected with an enhanced chemiluminescence (ECL) imaging system (Analytik Jena, Jena, Germany), and images were acquired with a ChemiDoc MP Imaging System (Bio-Rad, Hercules, CA, USA). All the gels were loaded with glyceraldehyde-3-phosphate dehydrogenase (GAPDH) as an internal loading control. Densitometric analysis was performed using Image Lab software (Bio-Rad, Hercules, CA, USA). Two retina samples from each group were pooled together for one experiment and each experiment was repeated independently three times.

### 2.11. Statistical Analysis

Analysis of statistical significance was performed in each group by the Kruskal–Wallis and Mann–Whitney U tests with Prism 7.0 software (GraphPad Software, San Diego, CA, USA, accession on 14 March 2021). Data are presented as the means ± standard deviation (SD). For all analyses, differences were considered statistically significant at *p* < 0.05.

## 3. Results

### 3.1. RTA 402 Treatment Increased the RGC Survival Rate

**Figure 2:**
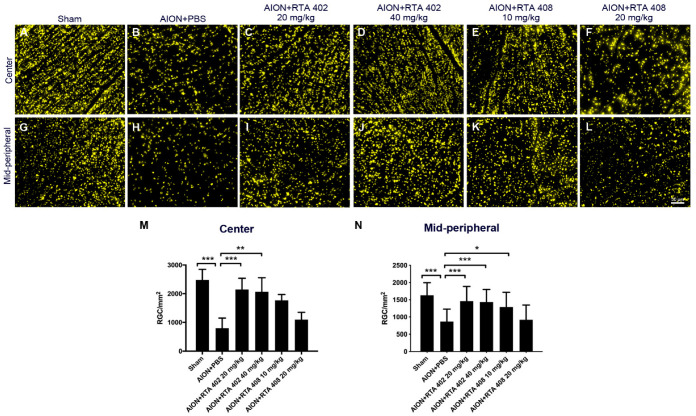
Retinal flat-mount and retinal ganglion cell (RGC) morphometry. (**A**–**L**) Representative flat-mount of retina after Fluoro-Gold (FG) labeling. (**A**,**G**) Sham operation; (**B**,**H**) AION and PBS retina; (**C**,**D**,**I**,**J**) AION and 20 mg/kg and 40 mg/kg of RTA 402 treatment; and (**E**,**F**,**K**,**L**) AION and 10 mg/kg and 20 mg/kg of RTA 408 treatment. (**M**,**N**) The morphometry of center retina after rAION induction, the densities of the sham, AION + PBS, AION + 20 mg/Kg RTA 402, AION + 40 mg/Kg RTA 402, AION + 10 mg/Kg RTA 408, and AION + 20 mg/Kg RTA 408 groups were 2477.8 ± 374.3, 797.4 ± 353.9 (32.6% survival), 2146.5 ± 391.4 (87.8% survival), 2066.1 ± 492.4 (84.5% survival) cells/mm^2^, 1768.2 ± 503.1 (72.3% survival), and 1096.6 ± 618.0 (44.8% survival) cells/mm^2^, respectively. The morphometry of mid-peripheral retina after rAION induction, the densities of the sham, AION + PBS, AION + 20 mg/Kg RTA 402, AION + 40 mg/Kg RTA 402, AION + 10 mg/Kg RTA 408, and AION + 20 mg/Kg RTA 408 groups were 1630.1 ± 366.9 cells/mm^2^, 865.7 ± 361.5 (53.1% survival) cells/mm^2^, 1459.5 ± 427.0 (89.5% survival) cells/mm^2^, 1459.5 ± 367.0 (87.9% survival) cells/mm^2^, 1288.9 ± 427.1 (79.1% survival) cells/mm^2^, and 918.2 ± 427.7 (56.3% survival) cells/mm^2^, respectively. (Scale bar = 50 μm, *n* = 6 in each group, and *, *p* < 0.05, **, *p* < 0.01, and ***, *p* < 0.001.).

FG is used for retrograde axonal tracer transport to label neurons [36]. After four weeks of rAION modeling, the densities of the RGCs in central retinas of the sham, AION + PBS, AION + 20 mg/Kg RTA 402, AION + 40 mg/Kg RTA 402, AION + 10 mg/Kg RTA 408, and AION + 20 mg/Kg RTA 408 groups were 2477.8 ± 374.3, 797.4 ± 353.9 (32.6% survival), 2146.5 ± 391.4 (87.8% survival), 2066.1 ± 492.4 (84.5% survival), 1768.2 ± 503.1 (72.3% survival), and 1096.6 ± 618.0 (44.8% survival) cells/mm^2^, respectively. The densities of the RGCs in the mid-peripheral retinas of the sham, AION + PBS, AION + 20 mg/kg RTA 402, AION + 40 mg/kg RTA 402, AION + 10 mg/kg RTA 408, and AION + 20 mg/kg RTA 408 groups were 1630.1 ± 366.9, 865.7 ± 361.5 (53.1% survival), 1459.5 ± 427.0 (89.5% survival), 1459.5 ± 367.0 (87.9% survival), 1288.9 ± 427.1 (79.1% survival), and 918.2 ± 427.7 (56.3% survival) cells/mm^2^, respectively (Figure 2). Notably, treatment with RTA 408 did not significantly rescue RGC survival after AION induction. The results indicate that RTA 402 treatment increases the survival rate of RGCs after ischemic injury.

### 3.2. RTA 402 Treatment Recused Visual Function

**Figure 3:**
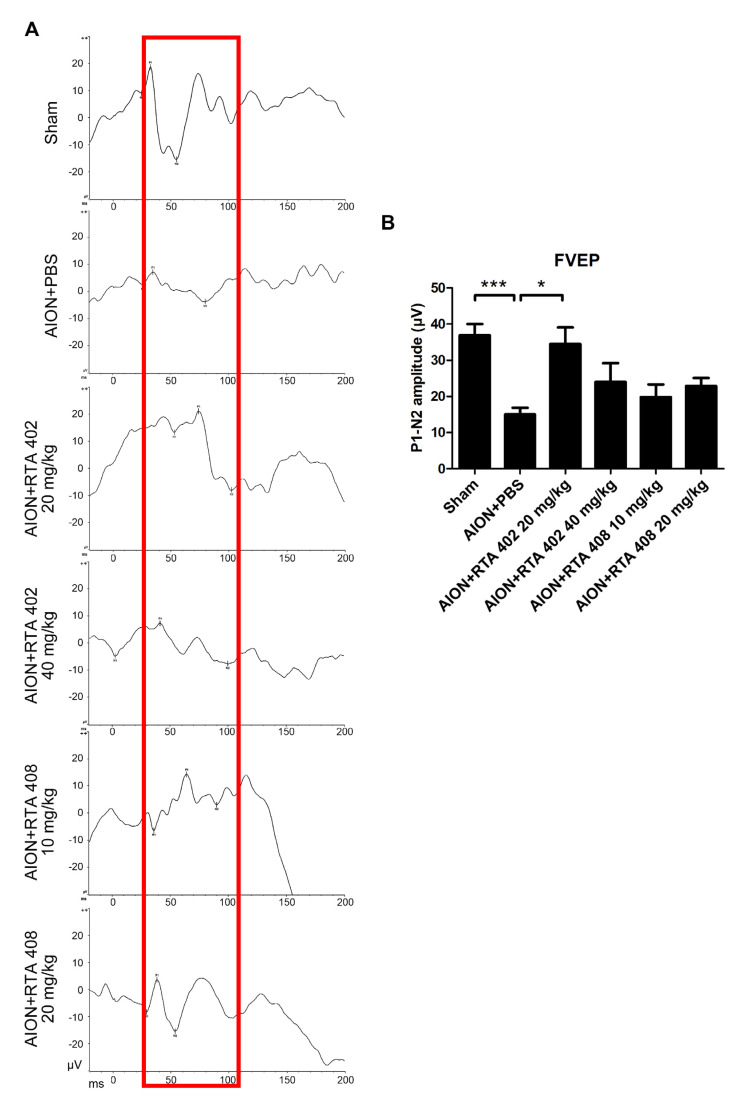
Flash VEPs. (**A**) The representative flash visual evoked potential (FVEPs) described 28 days after rAION induction. (**B**) The P1-N2 amplitudes of the sham, AION+PBS, AION+RTA 402 20 mg/kg, AION+RTA 402 40 mg/kg, AION+RTA 408 10 mg/kg, and RTA408 20 mg/kg groups were 36.88 ± 10.41 μV, 15.0 ± 5.82 μV, 34.51 ± 10.22 μV, 24.01 ± 10.34 μV, 19.8 ± 7.89 μV, and 22.91 ± 4.43 μV, respectively (Y-axis = 10 μV, X-axis = 50 ms. *n* = 6 in each group, *, *p* < 0.05, and ***, *p* < 0.001.).

FVEP plots were recorded 28 days after ischemia induction. The P1-N2 amplitudes of the sham, AION+PBS, AION+RTA 402 20 mg/kg, AION+RTA 402 40 mg/kg, AION+RTA 408 10 mg/kg, and RTA 408 20 mg/kg groups were 36.88 ± 10.41, 15.0 ± 5.82, 34.51 ± 10.22, 24.01 ± 10.34, 19.8 ± 7.89, and 22.91 ± 4.43 μV, respectively (Figure 3A,B). The FVEP results demonstrated that the 20 mg/kg RTA 402 treatment group had significantly preserved visual function compared to the PBS-treated group 28 days after the induction of ischemia. However, the RTA-408-treated groups did not show preservation of visual function. The RGC morphometry and visual function results suggest that a 20 mg/kg RTA 402 dose produces significant treatment effects. Based on these data, we proposed a 20 mg/kg dose of RTA 402 for further experiments.

### 3.3. RTA 402 Prevented Optic Nerve Edema and RNFL Atrophy

**Figure 4:**
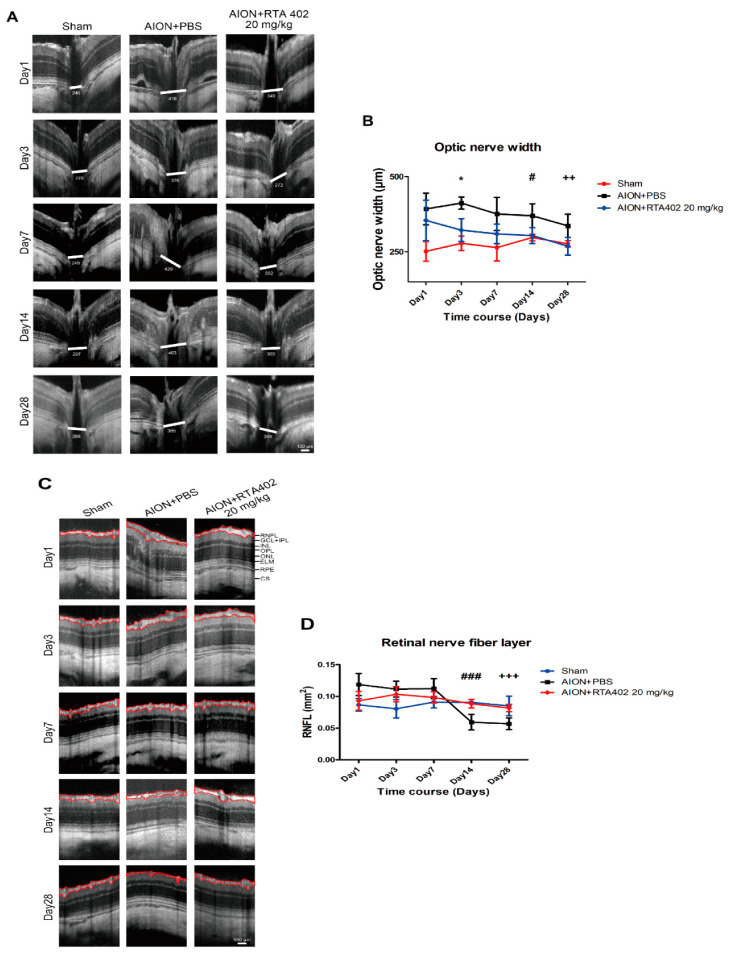
OCT images of ONW and RNFL. (**A**) The representative ONW described day 1, 3, 7, 4, and 28 after AION induction. (**B**) The ONW of the RTA-402-treated group represented the significant reduction in edema compared to the PBS-treated group at day 3, 14, and 28 (322.2 ± 38.2 μm versus 412.4 ± 20.18 μm, 304.06 ± 6.22 μm versus 370.0 ± 39.86 38.2 μm, and 268.7 ± 29.65 μm versus 336.6 ± 38.63 μm). (**C**) The representative RNFL described day 1, 3, 7, 14, and 28 after AION induction. (**D**) The RNFL of the RTA-402-treated group showed the significant prevention of RNFL thickness, compared to the PBS-treated group at day 14 and 28 (0.088 ± 0.007 mm^2^ versus 0.059 ± 0.012 mm^2^, 0.082 ± 0.006 mm^2^ versus 0.057 ± 39.86 0.009 mm^2^) (RNFL: retinal nerve fiber layer; GCL: ganglion cell layer; IPL: inner plexiform layer; INL: inner nuclear layer; ONL: outer nuclear layer; OPL: outer plexiform layer; ELM: external limiting membrane; RPE: retinal pigment epithelium; CS: choroidal stroma. Scale bar = 130 μm; *n* = 6 in each group; Day 3: *, *p* < 0.05; Day 14: #, *p* < 0.05, and ###, *p* < 0.001; Day 28: ++, *p* < 0.01, and +++, *p* < 0.001).

Acute inflammation induces optic nerve disc edema and macrophage infiltration, which cause optic nerve swelling and RNFL atrophy [19,37,38]. Spectral domain OCT was used to record the ONW and RNFL over time. The ONH immediately showed swelling 1 day after AION induction in both the RTA-402- and PBS-treated groups. There was a significant reduction in optic nerve edema at days 3, 14, and 28 after ischemic injury in the RTA-402-treated group compared to the PBS-treated group (Figure 4A,B). Furthermore, thickening of the RNFL was significantly prevented on days 14 and 28 after AION induction in the RTA-402-treated group compared to the PBS-treated group (Figure 4C,D). The recovery of the RNFL confirmed the survival rate of the RGCs, as observed by FG labeling. These results demonstrate that RTA 402 can reduce optic nerve swelling and thickening of the RNFL in the acute phase of inflammation after insult.

### 3.4. RTA 402 Treatment Decreased RGC Apoptosis in the Retina

**Figure 5:**
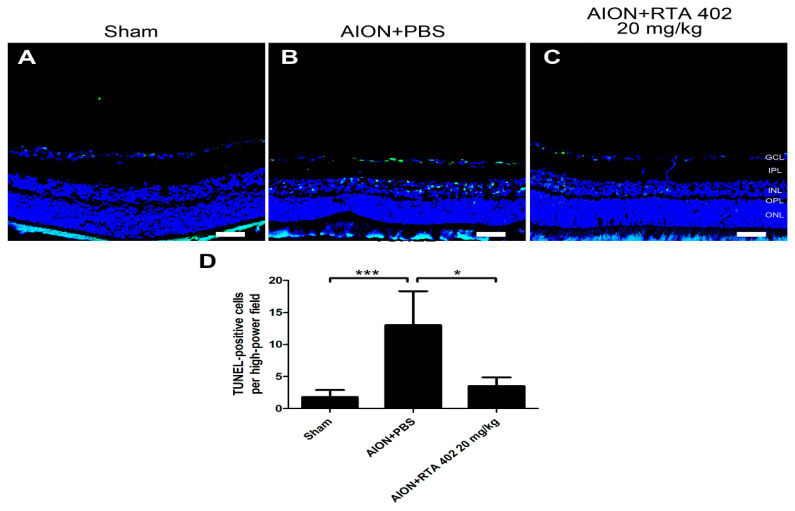
TUNEL assay in retinal cross-section. (**A**–**C**) The representative of retinal fields with Terminal-Deoxynucleotidyl-Transferase-Mediated Nick End Labeling (TUNEL) (green) in the sham, AION+PBS, and AION+RTA 402 20 mg/kg groups was described 28 days after rAION induction. (**D**) Analysis of TUNEL-positive cells in each group. The number of TUNEL-positive cells in the sham, AION+PBS, and AION+RTA 402 20 mg/kg groups was 1.8 ± 1.0, 13.0 ± 5.3, and 3.5 ± 1.4 TUNEL-positive cells/HPF, respectively (scale bar = 50 µm; *n* = 6 for each group; *, *p* < 0.05, and ***, *p* < 0.001. GCL: ganglion cell layer; IPL: inner plexiform layer; INL: inner nuclear layer; OPL: outer plexiform layer; ONL: outer nuclear layer).

To determine whether RTA 402 may protect RGCs from apoptosis, a TUNEL assay was performed on frozen retinal cross-sections. The numbers of TUNEL-positive cells in the sham, AION+PBS, and AION+RTA 402 20 mg/kg groups were 1.8 ± 1.0, 13.0 ± 5.3, and 3.5 ± 1.4 cells/HPF, respectively (Figure 5A–C). There was a significant decrease in the number of TUNEL-positive cells in the RTA 402-treated group compared to the PBS-treated group (Figure 4D). These results showed that RTA 402 had a significant antiapoptotic effect after ischemic induction.

### 3.5. RTA 402 Restrained Macrophage Infiltration in the Optic Nerve

**Figure 6:**
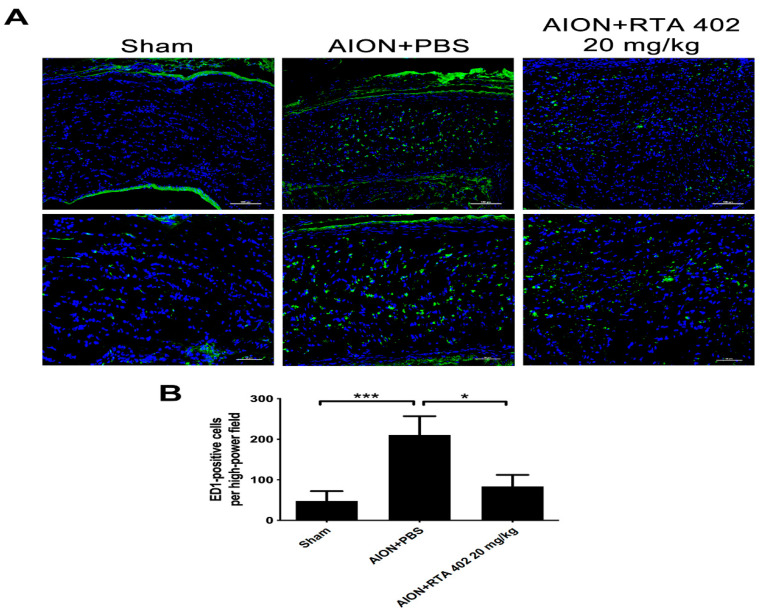
ED-1-positive cells infiltrate in the infarcted ON. (**A**) The representative of optic nerve fields with ED-1 (green) in the sham, AION+PBS, and AION+RTA 402 20 mg/kg groups were described 28 days after rAION induction. (**B**) Analysis of ED-1-positive cells in each group. The number of ED-1-positive cells in the sham, AION+PBS, and AION+RTA 402 20 mg/kg groups was 48.0 ± 24.07, 210.3 ± 46.54, and 83.56 ± 28.77 ED-1-positive cell/HPF, respectively (the upper column of scale bar = 100 µm, the lower column of scale bar = 50 µm, *n* = 6 in each group, * *p* < 0.05, *** *p* < 0.001).

The cellular immune response induces inflammatory compounds after rAION induction [31]. To confirm inflammation in the optic nerve after rAION model induction, ED-1, a macrophage marker in the CNS, was used [39,40]. After 28 days of ischemic optic neuropathy, the RTA-402-treated group showed less infiltration of ED-1-positive cells than the PBS-treated group (Figure 6). These results indicate that RTA 402 has an anti-inflammatory effect on optic nerve lesions.

### 3.6. RTA 402 Inhibited Anti-Inflammatory Cytokine Release and Increased Proinflammatory Protein Expression

**Figure 7:**
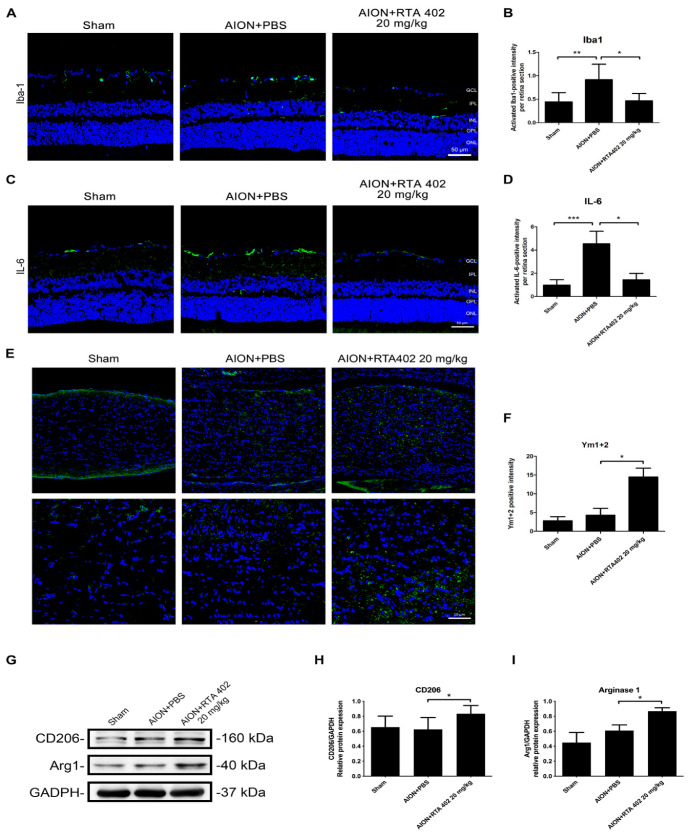
Immunofluorescence of Iba1, IL-6, and Ym1 + 2 in representative retina and optic nerve after AION induction. Immunoblot of Arg1 and CD206 in representative retina after AION induction. (**A**) The representative of retinal fields with Iba1 (green) in the sham, AION+PBS, and AION+RTA 402 20 mg/kg groups were described 28 days after rAION induction. (**B**) Analysis of Iba1-positive intensity in each group. The intensity in the sham, AION+PBS, and AION+RTA 402 20 mg/kg groups was 0.44 ± 0.2, 0.92 ± 0.33, and 0.47 ± 0.16 Iba1-positive intensity/HPF, respectively. (**C**) The representative of retinal fields with IL-6 (green) in the sham, AION+PBS, and AION+RTA 402 20 mg/kg groups was described 28 days after rAION induction. (**D**) Analysis of IL-6-positive intensity in each group. The intensity in the sham, AION+PBS, and AION+RTA 402 20 mg/kg groups were 0.98 ± 0.47, 4.55 ± 1.08, and 1.44 ± 0.55 IL-6-positive intensity/HPF, respectively. (**E**) The representative of optic nerve fields with Ym1 + 2 (green) in the sham, AION+PBS, and AION+RTA 402 20 mg/kg groups were described 28 days after rAION induction. (**F**) Analysis of Ym1 + 2-positive intensity in each group. The intensity in the sham, AION+PBS, and AION+RTA 402 20 mg/kg groups were 2.8 ± 1.09, 4.3 ± 1. 8, and 14.5 ± 2.35 Ym1 + 2-positive intensity/HPF, respectively. (**G**) Western blot images of Arg1and CD206 protein expression. (**H**,**I**) The quantitated data presented as the mean ± SD for independent experiments. (The scale bar of retina field = 50 µm, the upper column of scale bar in optic nerve field = 100 µm, and the lower column of scale bar in optic nerve field = 50 µm; *n* = 6 in each group; *, *p* < 0.05, **, *p* < 0.01, ***, *p* < 0.001. GCL: ganglion cell layer; IPL: inner plexiform layer; INL: inner nuclear layer; OPL: outer plexiform layer; ONL: outer nuclear layer.).

Neuroinflammation is widely stimulated by activated glial cells after ischemic injury in the CNS and immune cells infiltrate the brain, resulting in a series of neuronal stresses and the inducing of cell death [41]. Microglia, the first activated immune cells in the CNS, modulate inflammation in acute lesion areas [13]. IHC analysis showed that Iba1-positive cells were restricted to the retina after RTA 402 treatment (Figure 7A,B). The content of IL-6, a marker of proinflammatory proteins, decreased in the retina after RTA 402 treatment (Figure 7C,D), and there was an increase in Ym1 + 2 expression in the optic nerve after RTA 402 treatment (Figure 7E,F). Immunoblot analysis revealed increases in the protein expression of the M2 markers of inflammation, arginase 1 and the mannose receptor (CD206), in the RTA-402-treated group compared to the PBS-treated group (Figure 7G–I). These results show that RTA 402 can elevate inflammatory responses after AION induction.

### 3.7. RTA 402 Suppressed Demyelination after Ischemic Injury

**Figure 8:**
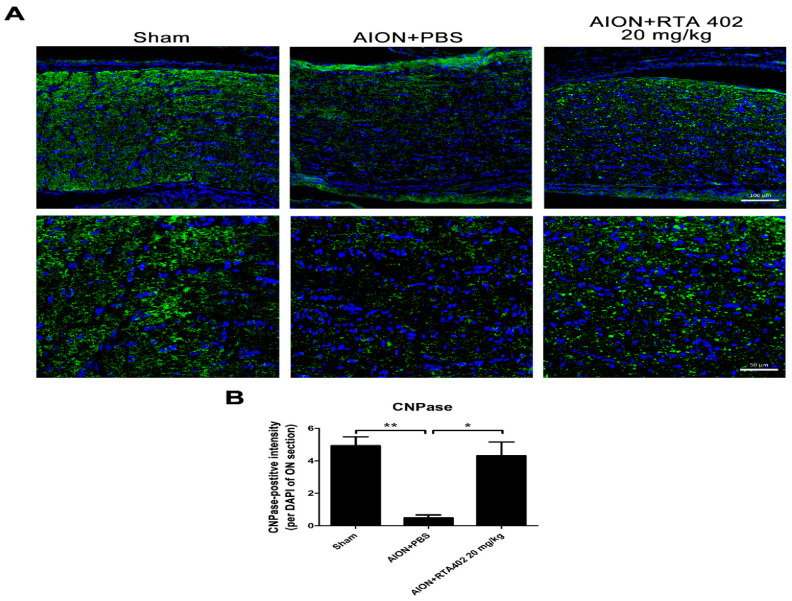
Immunofluorescence of CNPase in representative of optic nerve after AION induction. (**A**) The representative of retinal fields with CNPase (green) in the sham, AION+PBS, and AION+RTA 402 20 mg/kg groups were described 28 days after rAION induction. (**B**) Analysis of CNPase-positive intensity per DAPI in each group. The intensity in the sham, AION+PBS, and AION+RTA 402 20 mg/kg groups were 4.93 ± 0.55, 0.49 ± 0.19, and 4.32 ± 0.84 CNPase positive-intensity/DAPI, respectively. (The upper column of scale bar = 100 µm, the lower column of scale bar = 50 µm, *n* = 6 in each group; *, *p* < 0.05, **, *p* < 0.01.).

Optic nerve lesions cause substantial axonal damage to neurons and trigger cell apoptosis and demyelination in the optic nerve [42]. To investigate axonal damage after AION induction, CNPase was used to recognize the myelin sheath [43]. A lower CNPase-positive signal was observed in the optic nerve of the PBS-treated group than the RTA-402-treated group. Additionally, treatment in the RTA-402-treated group prevented demyelination after AION induction (Figure 8A,B). These data show that RTA 402 may have a demyelination effect during ischemic neuropathy.

### 3.8. RTA 402 Modulated the Nrf2 and NFκB Signaling Pathway to Prevent Oxidative Stress in AION Induction

**Figure 9:**
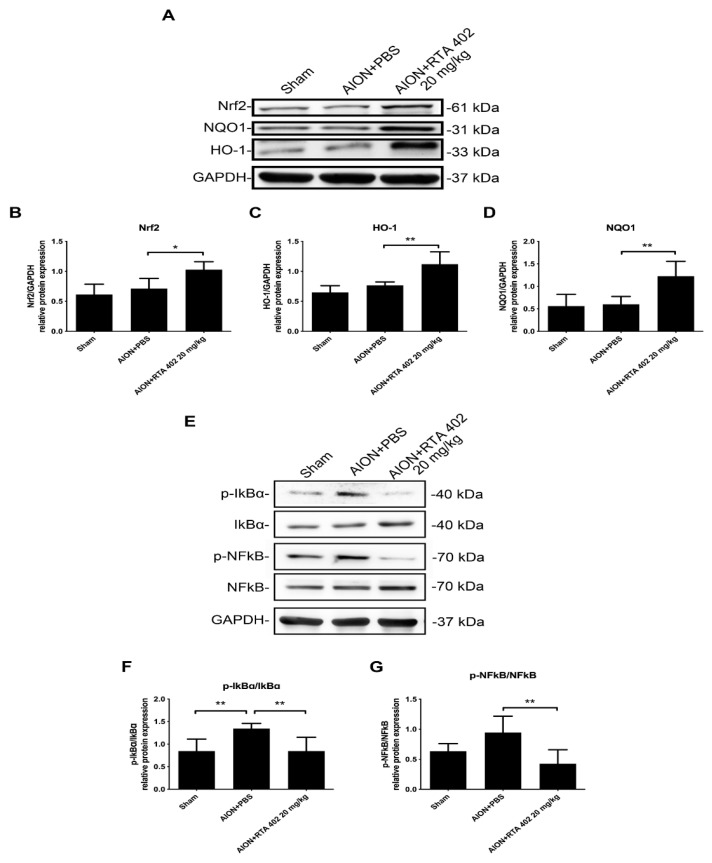
Activated Nrf2 and inhibited NFκB pathway in AION induction. (**A**) Western blot images of Nrf2, NQO1, and HO-1 protein expression. (**B**–**D**) The quantitated data of PBS-treated and RTA-402-treated groups presented as the mean ± SD for three independent experiments. (**E**) Western blot images of IκBα, *p*-IκBα, NFκB, and *p*-NFκB protein expression. (**F**,**G**) The quantitated data presented as the mean ± SD for three independent experiments. (*n* = 6 in each group; *, *p* < 0.05, ** *p* < 0.01).

Previous studies have shown that reactive oxygen species (ROS) induce neuronal damage after ischemic stroke. Nrf2 is a transcription factor involved in antioxidative protein modulation [19,44]. Immunoblot analysis demonstrated that the Nrf2 protein expression level significantly increased in the RTA-402-treated group compared to the PBS-treated group. The contents of the target proteins NQO1 and HO-1 were also significantly enhanced in the RTA-402-treated group (Figure 9A–D). Moreover, the RTA-402-treated group exhibited a decrease in the expression levels of *p*-IκBα and its downstream protein *p*-NFκB (Figure 9E–G). These results suggest that RTA 402 triggers the Nrf2 signaling pathway, represses IκBα phosphorylation, and suppresses NFκB activation to induce antioxidative stress after AION induction.

## 4. Discussion

In this work, we presented the neuroprotective results of RTA 402 in an rAION model, showed its effects against oxidative stress, the anti-inflammatory response, and found that the molecular signaling pathway was involved. The survival rate of RGCs and visual function, as demonstrated by FVEP measurements and FG labeling, were significantly better preserved in RTA-402-treated eyes than vehicle-treated eyes. These data confirmed that RTA 402 had a beneficial influence on eyes.

The immunoblot results demonstrated the significantly elevated expression of Nrf2, a transcription factor that binds to antioxidant response elements (AREs) and enhances the expression of NQO1 and HO-1 to protect against oxidative challenge [45]. ROS expression has been reported in retinal ischemia or hypoxia, causing the loss of visual function and irreversible neuronal and axonal damage [46]. A previous study confirmed that increasing Nrf2 protein expression maintained the viability of RGCs in an rAION model [19,34]. In addition, Nrf2 activators have been approved by the FDA as drugs to treat kidney disease and relapsing–remitting multiple sclerosis [47,48,49]. Our TUNEL assay results suggested that the administration of RTA 402 has an antiapoptotic effect on RGCs after rAION model induction. We also observed a significant increase in the levels of NQO1 and HO-1 via Nrf2 expression after RTA 402 treatment and AION induction. These observations suggest that improving the Nrf2 protein level may suppress oxidative stress, lead to RGC survival, and promote the antiapoptotic effects of RTA 402.

Previous research has documented that ED-1-positive cells are found during ischemic injury to the optic nerve [19,34], enhancing microglia/macrophage transfer to their activated forms in order to secrete inflammatory cytokines and activate inflammation [50]. However, overactivated microglia provide excessive amounts of the inflammatory ligands IL-6, IL-1β, and TNF-α, causing ROS, iNOS, NFκB, and NLRP3 inflammatory complex activation; additionally, disruption of neuronal homeostasis was observed in ischemic optic neuropathy [11,51,52]. Our immunoblot and IHC results indicate a decrease in IL-6- and Iba1-positive cell accumulation and inhibition of NFκB and IκBα phosphorylation in the retina after the administration of RTA 402. In addition, significant expression levels of the anti-inflammatory factors TGF-β and Ym1 + 2 were observed in the retina and optic nerve after RTA 402 treatment. TGF-beta can be a prosurvival factor, generally, only in acute damage models [53,54]. However, several pieces of evidence provide hints of detrimental action of TGF-beta in animal and human diabetic retinas [55], given the fibrotic activity of the growth factors. Moreover, the Nrf2-dependent signaling pathway can mediate the immune response during cellular metabolism, shift the cells to an anti-inflammatory phenotype, and release the anti-inflammatory factors Arg-1, CD206, IL-10, and TGF-β [56,57]. These phenomena might confirm that RTA 402 can trigger Nrf2, modulate M1/M2 microglial polarization, stimulate microglia toward the M2 phenotype, release anti-inflammatory cytokines, and shift to an immunosuppressive state.

Axonal demyelination was observed during optic nerve injury caused by inflammation and induced the loss of RGC axonal function [3,58,59]. RGC axonal damage often causes axonal degeneration and causes permanent loss to the cell body via apoptosis. We directly revealed axonal myelination by labeling CNPase, an oligodendrocyte marker in the CNS [42,60,61]. Our observations might confirm that RTA 402 rescues axonal myelination in RGCs during ischemic optic injury. However, two things remain to be discussed in the future. First, the RTA 402 modulated when it initially was injected immediately after rAION induction. Administration several days after the rAION induction needs to be studied. Second, the rodent model responses may or may not be relevant with respect to primate responses.

## 5. Conclusions

**Figure 10:**
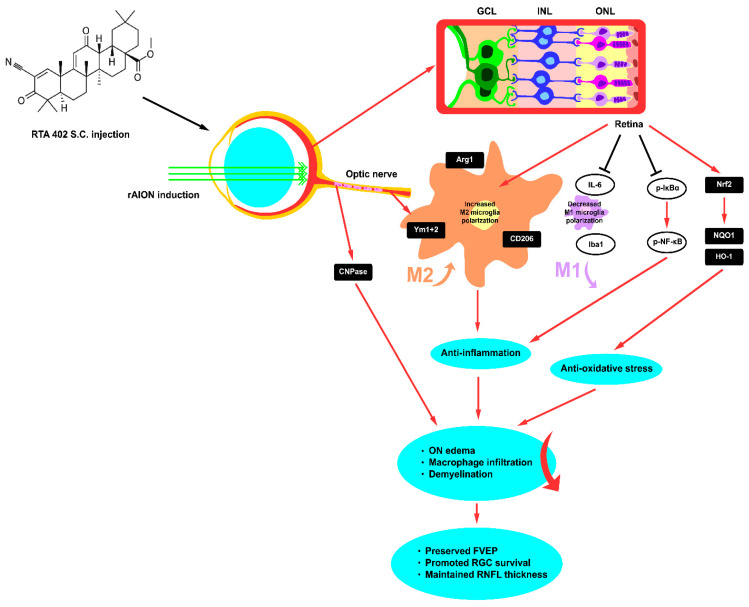
Schematic diagram of neuroprotection effect of RTA 402 in rAION model. After AION induction, RTA 402 treatment indicates the antiapoptotic and antioxidative stress. RTA 402 rescues the RGCs and visual function by modulating the Nrf2 signaling pathway and promoting M2 microglia polarization by decreasing IL-6 and Iba1 and increasing Ym1 + 2 expression in ischemic optic neuropathy. RTA 402 prevents optic nerve edema and maintains axonal myelination as well as RLFL thickness.

In conclusion, our results demonstrate that RTA 402 had a remarkably neuroprotective effect by preserving visual function and RGC survival, inhibiting apoptosis, restraining oxidative stress, shifting microglia to an anti-inflammatory phenotype, and maintaining axonal myelination via the Nrf2 signaling pathway in the retina (Figure 10). Together, our data indicate the potential therapeutic application of RTA 402 for the protection of RGCs against apoptotic death in ischemic optic neuropathy.

## Figures and Tables

**Table 1:** Summary of the rats used in study.

	Sham	AION + PBS	AION + RTA 402 (20 mg/kg)	AION + RTA 402 (40 mg/kg)	AION + RTA 408 (10 mg/kg)	AION + RTA 408 (20 mg/kg)
FG retrograde labeling	6	6	6	6	6	6
OCT, VEP, TUNEL, and IHC	6	6	6	6	6	6
Immunoblotting analysis	6	6	6	6	6	6

## Data Availability

All data generated or analyzed during this study are included in this article.
